# CREB3L2-mediated expression of Sec23A/Sec24D is involved in hepatic stellate cell activation through ER-Golgi transport

**DOI:** 10.1038/s41598-017-08703-6

**Published:** 2017-08-11

**Authors:** Shotaro Tomoishi, Shinichi Fukushima, Kentaro Shinohara, Toshiaki Katada, Kota Saito

**Affiliations:** 1 Department of Physiological Chemistry, Graduate School of Pharmaceutical Sciences, University of Tokyo, 7-3-1 Hongo, Bunkyo-ku, Tokyo 113-0033 Japan; 2 Faculty of Pharmacy, Musashino University, Tokyo, 202-8585 Japan

## Abstract

Hepatic fibrosis is caused by exaggerated wound healing response to chronic injury, which eventually leads to hepatic cirrhosis. Differentiation of hepatic stellate cells (HSCs) to myofibroblast-like cells by inflammatory cytokines is the critical step in fibrosis. This step is accompanied by enlargement of the endoplasmic reticulum (ER) and Golgi apparatus, suggesting that protein synthesis and secretion are augmented in the activated HSCs. However, the process of rearrangement of secretory organelles and their functions remain to be fully elucidated. Here, we revealed that differentiation alters early secretory gene expression. We observed significant isoform-specific upregulation of the inner coat protein complex II (COPII) components, Sec23A and Sec24D, via the transmembrane bZIP transcription factor, CREB3L2/BBF2H7, during HSC activation. Moreover, knockdown of these components abrogated the activation, suggesting that Sec23A/Sec24D-mediated ER to Golgi trafficking is required for HSC activation.

## Introduction

Hepatic fibrosis is considered as an exaggerated wound healing process in response to chronic liver injury, which is characterized by excessive extracellular matrix production. Advanced hepatic fibrosis leads to hepatic cirrhosis and eventually hepatocellular carcinoma^1^. Activation of hepatic stellate cells (HSCs) is mainly responsible for the progression of hepatic fibrosis. Although HSCs are quiescent cells storing vitamin A in normal liver, once activated by inflammatory cytokines, HSCs become highly proliferative, and differentiate into myofibroblast-like cells characterized by α-smooth muscle actin (α-SMA) expression and enhanced collagen I secretion^2, 3^. The differentiation is accompanied by enlargement of the endoplasmic reticulum (ER) and Golgi apparatus, suggesting that protein synthesis and secretion are enhanced in the activated HSCs^4, 5^. These enlargements of secretory organelles should alter the expression of early secretory gene components, however, this process remains to be fully elucidated.

Newly synthesized secretory proteins exit the ER via coat protein complex II (COPII)-coated vesicles to the Golgi. The formation of COPII vesicles has been well characterized^6^. The activated small GTPase Sar1 is recruited to the ER membrane, where it enhances the assembly of a pre-budding complex consisting of inner-coat complex Sec23/Sec24 and cargo receptors. When cargoes are captured by cargo receptors, the pre-budding complex interacts with outer-coat complex Sec13/Sec31, which enhances the hydrolysis of Sar1 to complete the vesicle formation^6^. Human cells express two isoforms of Sec23 and four isoforms of Sec24, which are thought to provide diversity to cargo recognition.

CREB3L2/BBF2H7 is a transmembrane transcription factor synthesized in the ER^7^. It has been reported in mice and fish that this transcription factor is involved in collagen secretion by directly binding to the promoter regions of *Sec23A* and *TANGO1* ^8–10^. In addition, the expression of several factors involved in ER-to-Golgi transport is decreased in a zebrafish mutant (*feelgood*) and in CREB3L2/BBF2H7-knockout medaka fish^8, 9^. As described above, CREB3L2/BBF2H7 is critical for skeletogenesis, however, its involvement in HSC activation remains to be investigated.

Here, we analysed the changes in early secretory gene expression in HSCs between the quiescent and activated states. Although the expression of most of the tested genes was reduced or unchanged upon activation, we observed isoform-specific upregulation of Sec23A and Sec24D. Interestingly, the expression of Sec24D was regulated by CREB3L2/BBF2H7.

Moreover, depletion of Sar1, Sec23A, Sec24D or CREB3L2/BBF2H7 hindered transforming growth factor β (TGF-β)-mediated HSC activation, suggesting that COPII-mediated transport via Sec23A/Sec24D is required for HSC activation.

## Results

### Sec23A and Sec24D are upregulated during primary rat HSC activation in an isoform-specific manner

**Figure 1:**
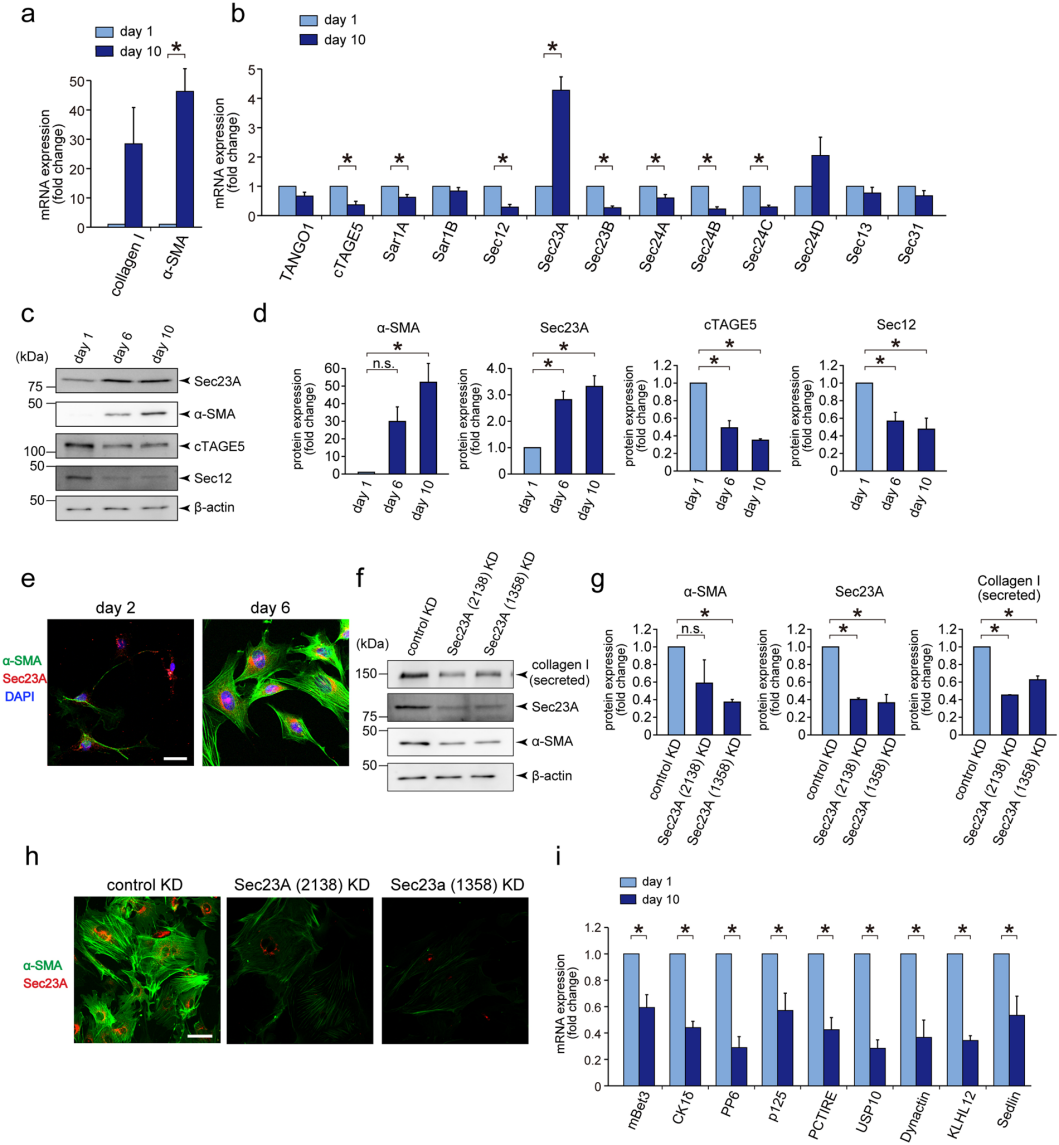
Sec23A is required for *in vitro* activation of primary rat HSCs. (**a** and **b**) Primary rat HSCs isolated by *in situ* perfusion were cultured for 1 to 10 days. Cells were collected for RNA extraction, followed by qRT-PCR (*n* = 5). (**c** and **d**) Primary rat HSCs isolated by *in situ* perfusion were cultured for 1, 6 and 10 days. Proteins were extracted and analysed by SDS-PAGE, followed by western blotting with anti-Sec23A, anti-α-SMA, anti-cTAGE5, anti-Sec12 and anti-β-actin antibodies. (**c**) Representative immunoblots. (**d**) Quantification of immunoblots (*n* = 3). The band intensities were normalized to those of β-actin. (**e**) Primary rat HSCs isolated by *in situ* perfusion were cultured for 2 to 6 days. Cells were fixed and stained with anti-Sec23A and α-SMA antibodies, and DAPI. Bars, 25 μm. (**f** and **g**) Primary rat HSCs isolated by *in situ* perfusion were cultured for 2 days and transfected with the indicated siRNA(s). After 4 days, whole cell proteins were extracted and analysed by SDS-PAGE, followed by western blotting with anti-Sec23A, anti-α-SMA and anti-β-actin antibodies. Medium was collected and the proteins were precipitated for SDS-PAGE, followed by western blotting with anti-collagen I antibody. (**f**) Representative immunoblots. (**g**) Quantification of immunoblots (*n* = 3). The band intensities were normalized to those of β-actin. (**h**) Primary rat HSCs isolated by *in situ* perfusion were cultured for 2 days and transfected with the indicated siRNA(s). After 4 days, cells were fixed and stained with anti-Sec23A and anti-α-SMA antibodies. Bars, 50 μm. (**i**) Primary rat HSCs isolated by *in situ* perfusion were cultured for 1 to 10 days. Cells were collected for RNA extraction and qRT-PCR (*n* = 5). Error bars represent mean ± SEM. *P < 0.05. Uncropped original blots were shown in Fig. S3.

To identify early secretory genes involved in HSC activation, we compared the transcription levels of secretory components between the quiescent and activated states. Primary rat HSCs isolated by *in situ* perfusion were cultured for 1–10 days for *in vitro* activation. As previously reported, HSCs cultured for 10 days showed significantly higher expression of collagen I and α-SMA compared with the 1-day-cultured cells, indicating that the cells differentiated into activated myofibroblasts (Fig. 1a)^11^. The differentiation of HSCs was also evident by cell morphology and by immunofluorescence staining of α-SMA (Fig. 1e). We then analysed the expression of early secretory genes. The gene expression of most of the COPII components including cTAGE5 and TANGO1, which have been identified as collagen cargo receptors^12–22^, did not change or even decreased upon HSC activation (Fig. 1b). Interestingly, Sec23A was upregulated during HSC activation in an isoform-specific manner (Fig. 1b). Sec24D was also upregulated, although this result was not statistically significant (Fig. 1b). We then verified these expression profiles at the protein level by western blotting and immunofluorescence. Although the expression of cTAGE5 and Sec12 decreased upon HSC differentiation, Sec23A expression increased together with α-SMA expression, a marker of HSC activation (Fig. 1c–e). These data suggest a potential role for Sec23A and Sec24D in HSC activation.

### Sec23A is required for primary rat HSC activation

To elucidate whether Sec23A is involved in HSC activation, we transfected the cells with siRNAs against *Sec23A* and checked the activation status. To minimize the risk of misinterpretation due to off-target effects, we used two individual siRNAs targeting different sequences. As shown in Fig. 1f and g, Sec23A knockdown efficiently reduced the expression of α-SMA. Interestingly, collagen I secretion to the medium was also reduced upon Sec23A knockdown (Fig. 1f and g). Reduced stress fibre formation was also evident by immunostaining with anti-α-SMA, although the cell morphology did not seem to change after the knockdown treatment (Fig. 1h). As collagen I and α-SMA are major markers of HSC activation, these findings indicate that Sec23A is necessary for the activation of primary HSCs. Sec23A is an inner COPII-coat component, which is known to interact with several effector molecules to achieve its functions^23^, hence, we checked whether Sec23A-interacting proteins are upregulated during HSC activation. The expression of all Sec23A-interacting proteins tested was reduced (Fig. 1i), implying that Sec23A and Sec24D upregulation may not exert its influence on HSC activation through Sec23A-interacting proteins.

### Sec23A and Sec24D are required for the activation of HSC cell line LX-2

**Figure 2:**
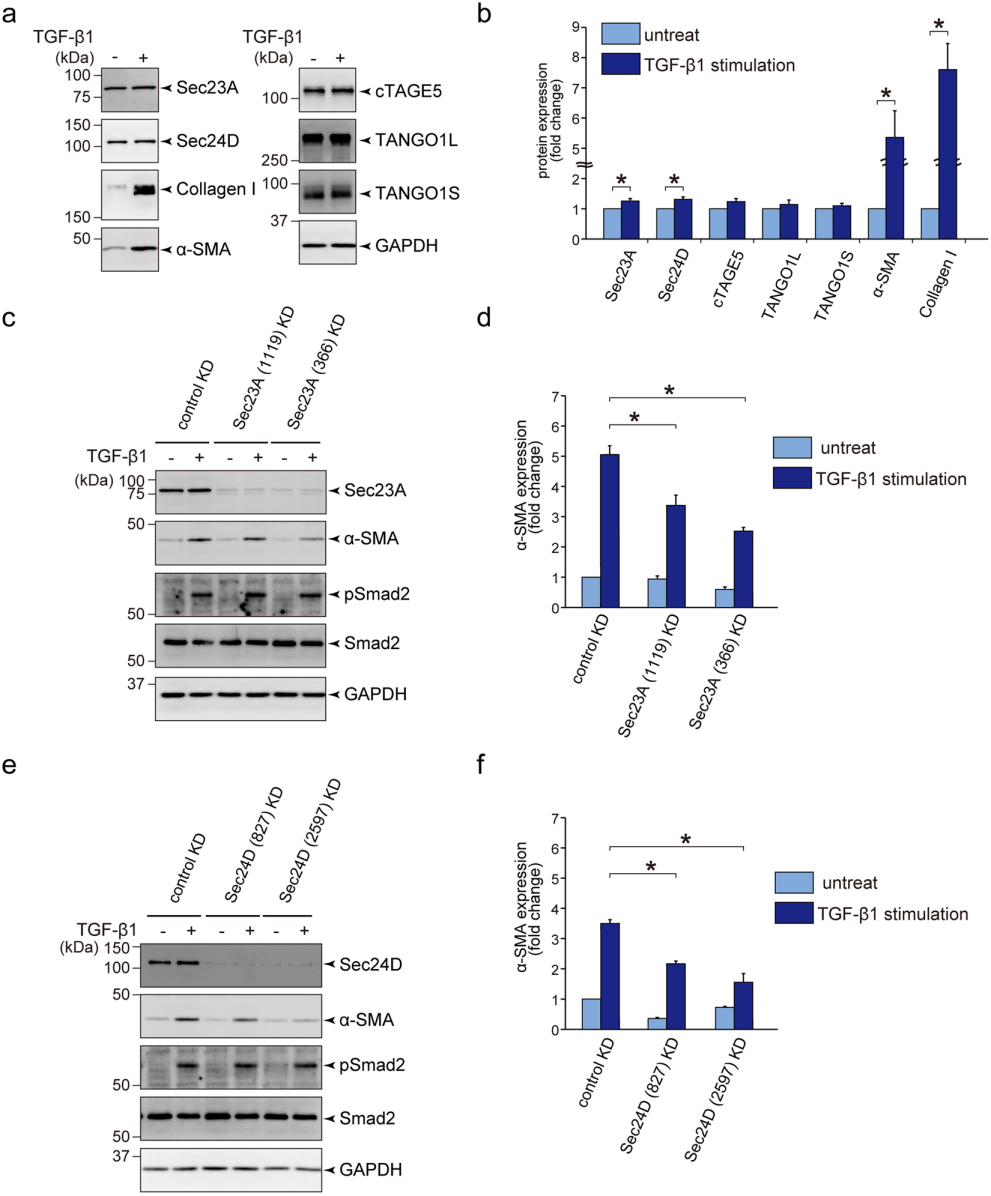
Sec23A and Sec24D are required for activation of LX-2 cells upon TGF-β1 stimulation. (**a** and **b**) LX-2 cells starved for 24 h with DMEM supplemented with 0.5% FBS were untreated or treated with 1 ng/ml TGF-β1 and cultured for 3 days. Cells were extracted and subjected to SDS-PAGE followed by western blotting with anti-Sec23A, anti-Sec24D, anti-collagen I, anti-α-SMA, anti-cTAGE5, anti-TANGO1 and anti-GAPDH antibodies. (**a**) Representative immunoblots. (**b**) Quantification of immunoblots (*n* = 6). The band intensities were normalized to those of GAPDH. (**c**–**f**) LX-2 cells transfected with the indicated siRNA(s) were cultured for 24 h in DMEM supplemented with 10% FBS. After starvation for 24 h with DMEM supplemented with 0.5% FBS, the cells were untreated or treated with 1 ng/ml TGF-β1 and cultured for 3 days. Proteins were extracted and subjected to SDS-PAGE, followed by western blotting with anti-Sec23A (**c**), anti-Sec24D (**e**), anti-α-SMA, anti-Smad2, anti-pSmad2 and anti-GAPDH antibodies. (**c**,**e**) Representative immunoblots. (**d**,**f**) Quantification of immunoblots (*n* = 3). The band intensities of α-SMA were normalized to those of GAPDH. Error bars represent mean ± SEM. *P < 0.05. Uncropped original blots were shown in Fig. S3.

To further elucidate the signalling pathway involved in HSC activation, we used human hepatic stellate cell line LX-2, which has been extensively characterized as a model for studying signalling molecules during activation by TGF-β^24^. As shown previously, TGF-β treatment efficiently enhanced the expression of collagen I and α-SMA in LX-2 cells^24^. Although the expression of cTAGE5, TANGO1L and TANGO1S did not change, we observed marginal but significant upregulation of Sec23A and Sec24D upon TGF-β treatment (Fig. 2a and b, and Fig. S1). We then checked whether knockdown of these proteins affects the activation of LX-2 cells. SiRNA-mediated depletion of Sec23A or Sec24D efficiently reduced the TGF-β-induced expression of α-SMA (Fig. 2c–f), suggesting that Sec23A and Sec24D are required for the activation of LX-2 cells. We then tested whether TGF-β transduces signals through Smad proteins in Sec23A or Sec24D-depleted cells. As shown in Fig. 2c and e, Smad2 was phosphorylated upon TGF-β stimulation to the same extent in depleted cells and in control cells. These results indicate that the reduced expression of α-SMA in Sec23A or Sec24D-depleted cells was probably not due to the impairment of TGF-β receptor signalling in LX-2 cells.

### CREB3L2/BBF2H7 controls the activation of LX-2 cells by transcriptionally regulating Sec23A and Sec24D

**Figure 3:**
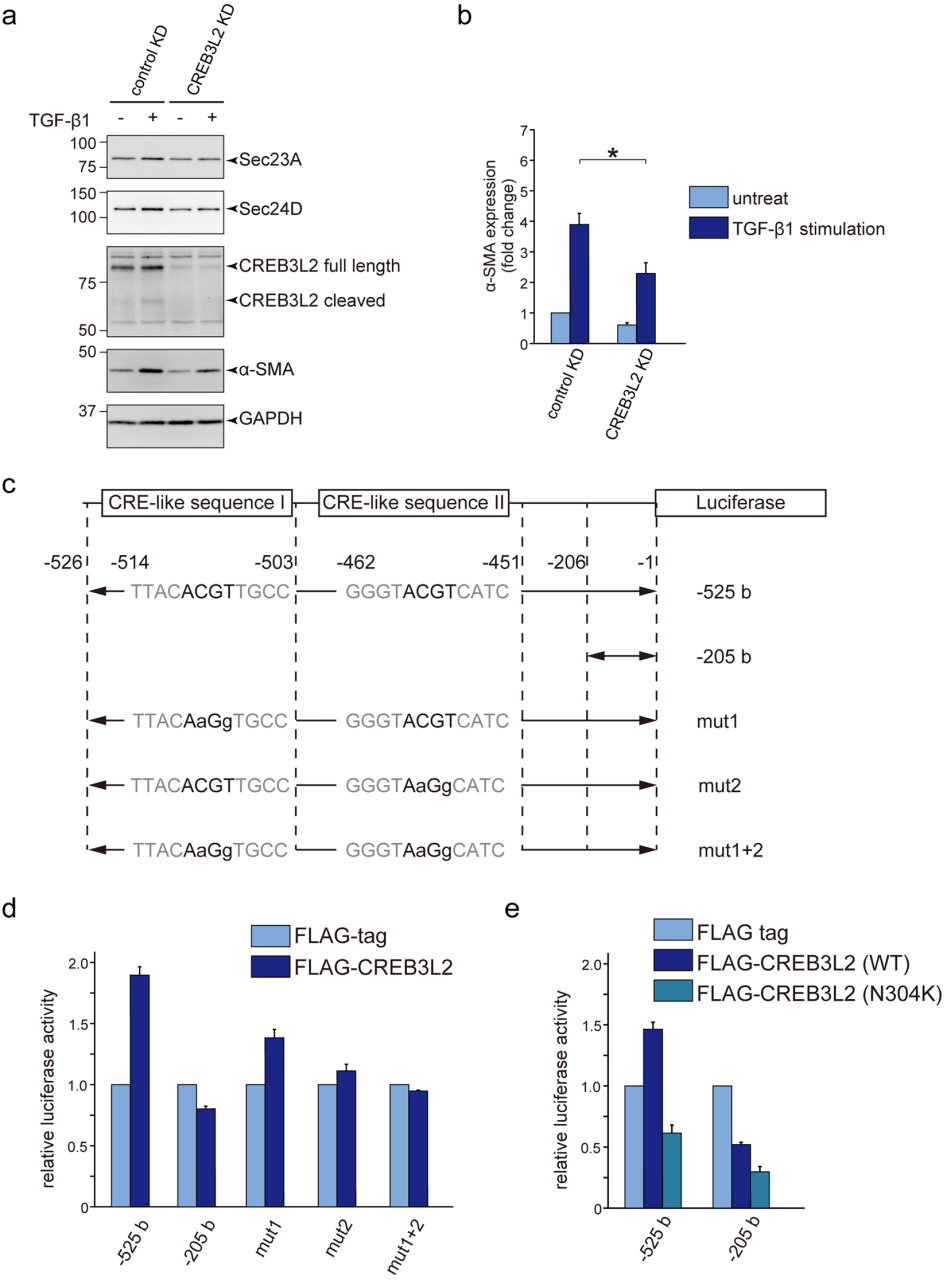
CREB3L2/BBF2H7 is involved in the activation of LX-2 cells by transcriptionally regulating Sec23A and Sec24D. (**a** and **b**) LX-2 cells transfected with the indicated siRNA(s) were cultured for 24 h in DMEM supplemented with 10% FBS. After starvation for 24 h with DMEM supplemented with 0.5% FBS, the cells were untreated or treated with 1 ng/ml TGF-β1 and cultured for 3 days. Proteins were extracted and subjected to SDS-PAGE, followed by western blotting with anti-Sec23A, anti-Sec24D, anti-CREB3L2/BBF2H7, anti-α-SMA and anti-GAPDH antibodies. (**a**) Representative immunoblots. (**b**) Quantification of immunoblots (*n* = 6). The band intensities of α-SMA were normalized to those of GAPDH. (c and d) Dual-luciferase reporter assay was performed with *Sec24D* promoter constructs attached to a luciferase gene and CREB3L2/BBF2H7 construct. (**c**) Schematic view of the reporter constructs. (**d** and **e**) Quantification of relative luciferase activity (*n* = 3). Error bars represent mean ± SEM. *P < 0.05. Uncropped original blots were shown in Fig. S3.

Because Sec23A has been identified as a target of transcription factor CREB3L2/BBF2H7 in chondrocytes and dermal fibroblasts^10, 25^, we tested whether LX-2 cell activation by Sec23A is affected by this transcription factor. CREB3L2/BBF2H7 knockdown abrogated the TGF-β-induced upregulation of Sec23A, suggesting that CREB3L2/BBF2H7-mediated Sec23A induction is involved in HSC activation (Fig. 3a). Interestingly, Sec24D upregulation was also inhibited by CREB3L2/BBF2H7 depletion (Fig. 3a). In accordance with the reduced expression of Sec23A and Sec24D, the TGF-β-induced α-SMA expression decreased upon CREB3L2/BBF2H7 knockdown (Fig. 3a and b).

Our observation that Sec24D expression depends on CREB3L2/BBF2H7 led us to check whether Sec24D is a target of CREB3L2/BBF2H7. To this end, we searched for a cAMP-responsive element (CRE)-like sequence within the *Sec24D* promoter and found five candidate sequences between 525 bp and 205 bp upstream of the *Sec24D* transcription start site (Fig. 3c). We then performed reporter assays. In cells with the 525-bp reporter construct, the reporter activity was significantly induced by the expression of FLAG-CREB3L2/BBF2H7 compared with FLAG-tag only (Fig. 3d). In contrast, the 205-bp reporter construct did not induce reporter activity after CREB3L2/BBF2H7 expression (Fig. 3d), indicating that Sec24D is transcriptionally regulated by CREB3L2/BBF2H7 and its responsive element lies between 525 bp and 205 bp upstream of the *Sec24D* transcription start site. In the zebrafish *feelgood* line, a missense mutation in the DNA-binding basic motif of CREB3L2/BBF2H7 has been reported to impair the function of the transcription factor, leading to defects in skeletal morphogenesis^9^. Of note, this mutant has also been reported to reduce the expression of Sec23A and Sec24D^9^. To check the specificity of CREB3L2/BBF2H7-mediated Sec24D expression, we introduced a corresponding mutation into human CREB3L2/BBF2H7 (N304K) and checked the effect by reporter assays. As shown in Fig. 3e, the N304K mutation failed to activate the transcription of Sec24D, further confirming the specificity of CREB3L2/BBF2H7 toward Sec24D. We then introduced mutations into the candidate CRE-like sequences (Fig. 3c). Interestingly, mutations in two of the CRE-like sequences (−514 to −503 bp and −462 to −451 bp) significantly decreased the induction of reporter activity (Fig. 3c and d). Moreover, when the mutations were introduced into both sequences, the increase in reporter activity was completely abrogated (Fig. 3c and d). Taken together, we concluded that Sec24D and Sec23A are transcriptionally regulated by CREB3L2/BBF2H7 during HSC activation.

### COPII-mediated transport, but not secretion to the extracellular milleu is required for HSC activation

**Figure 4:**
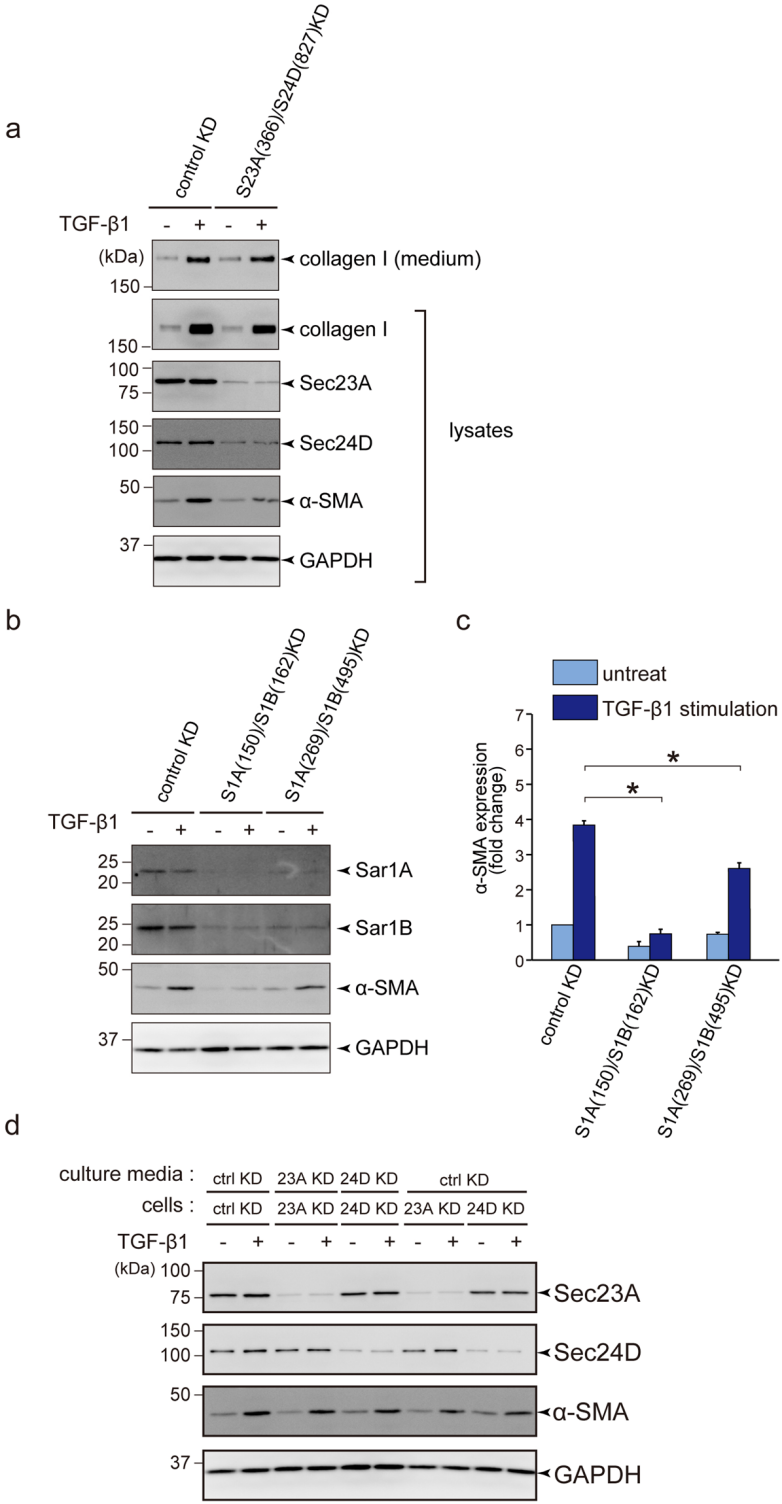
COPII-mediated ER to Golgi transport is required for activation of LX-2 cells upon TGF-β1 stimulation. (**a**) LX-2 cells transfected with the indicated siRNA(s) were cultured for 24 h in DMEM supplemented with 10% FBS. After starvation for 24 h with DMEM supplemented with 0.5% FBS, the cells were untreated or treated with 1 ng/ml TGF-β1 and cultured for 3 days. Proteins were extracted and subjected to SDS-PAGE, followed by western blotting with anti-collagen I, anti-Sec23A, anti-Sec24D, anti-α-SMA and anti-GAPDH antibodies (lysates). Medium was collected for SDS-PAGE followed by western blotting with anti-collagen I antibody (medium). Shown is a representative immunoblot analysis (*n* = 3). (**b** and **c**) LX-2 cells transfected with the indicated siRNA(s) were cultured for 24 h in DMEM supplemented with 10% FBS. After starvation for 24 h with DMEM supplemented with 0.5% FBS, the cells were untreated or treated with 1 ng/ml TGF-β1 and cultured for 3 days. Proteins were extracted and subjected to SDS-PAGE followed by western blotting with anti-Sar1A, anti-Sar1B, anti-α-SMA and anti-GAPDH antibodies. (**b**) Representative immunoblots. (**c**) Quantification of immunoblots (*n* = 3). The band intensities of α-SMA were normalized to those of GAPDH. (**d**) LX-2 cells transfected with the indicated siRNA(s) were cultured for 24 h in DMEM supplemented with 10% FBS. After starvation for 24 h with DMEM supplemented with 0.5% FBS, the cells were untreated or treated with 1 ng/ml TGF-β1 for 24 h. The culture medium was replaced with medium incubated with cells treated with the indicted siRNA(s) and further cultured for 48 h. Proteins were extracted and subjected to SDS-PAGE, followed by western blotting with anti-Sec23A, anti-Sec24D, anti-α-SMA and anti-GAPDH antibodies. Error bars represent mean ± SEM. *P < 0.05. Uncropped original blots were shown in Fig. S3.

Several reports have indicated that both Sec23A and Sec24D are important for collagen I secretion^17^. In addition, collagen I secretion has been shown to be involved in HSC activation^26, 27^. Thus, we tested whether depletion of Sec23A and Sec24D from LX-2 cells impairs collagen I secretion. Surprisingly, even though knockdown of both Sec23A and Sec24D efficiently reduced the expression of α-SMA, secretion and intracellular accumulation of collagen I were unchanged compared with the control cells with or without TGF-β stimulation (Fig. 4a). These data imply that it is not a defect in collagen I secretion that inactivates HSCs upon Sec23A/Sec24D depletion. As Sec23A/Sec24D serves as an inner-coat complex essential for COPII-vesicle formation, we next verified whether COPII-mediated transport is involved in HSCs activation. We knocked down Sar1A and Sar1B, small GTPases required for COPII-vesicle formation, and checked the HSC activation status. As shown in Fig. 4b and c, depletion of both Sar1A and Sar1B efficiently reduced α-SMA induction upon TGF-β stimulation, indicating that COPII-mediated ER to Golgi transport is required for HSC activation.

The fact that Sec23A and Sec24D form a pre-budding complex with cargo receptors prompted us to consider the possibility that Sec23A and Sec24D are responsible for trafficking of key molecule(s) other than collagen I, which may be critical for the activation of HSCs. To elucidate whether the key factor(s) is secreted from the cell surface or stays within the cells, cells depleted of either Sec23A or Sec24D were incubated with medium that was pre-cultured with control cells. If HSC activation depends on a secreted factor, then the cells depleted of either Sec23A or Sec24D should be efficiently activated by medium containing the key factor for HSC activation. When cells treated with the control siRNA were incubated with the medium from control cells, α-SMA was efficiently induced upon TGF-β treatment (Figs 4d and S2, lanes 1 and 2). In contrast, when cells depleted of either Sec23A or Sec24D were incubated with medium pre-cultured with depleted cells, α-SMA induction was significantly reduced (Figs 4d and S2, lanes 3–6). Even when medium from control cells was introduced to the cells depleted of COPII proteins, α-SMA induction remained inhibited (Figs 4d and S2, lanes 7–10). These results indicate that Sec23A/Sec24D-mediated COPII transport, but not secretion to the extracellular milleu, is required for HSC activation.

### CREB3L2/BBF2H7 relocation from the ER to the Golgi does not depend on Sec23A and Sec24D

**Figure 5:**
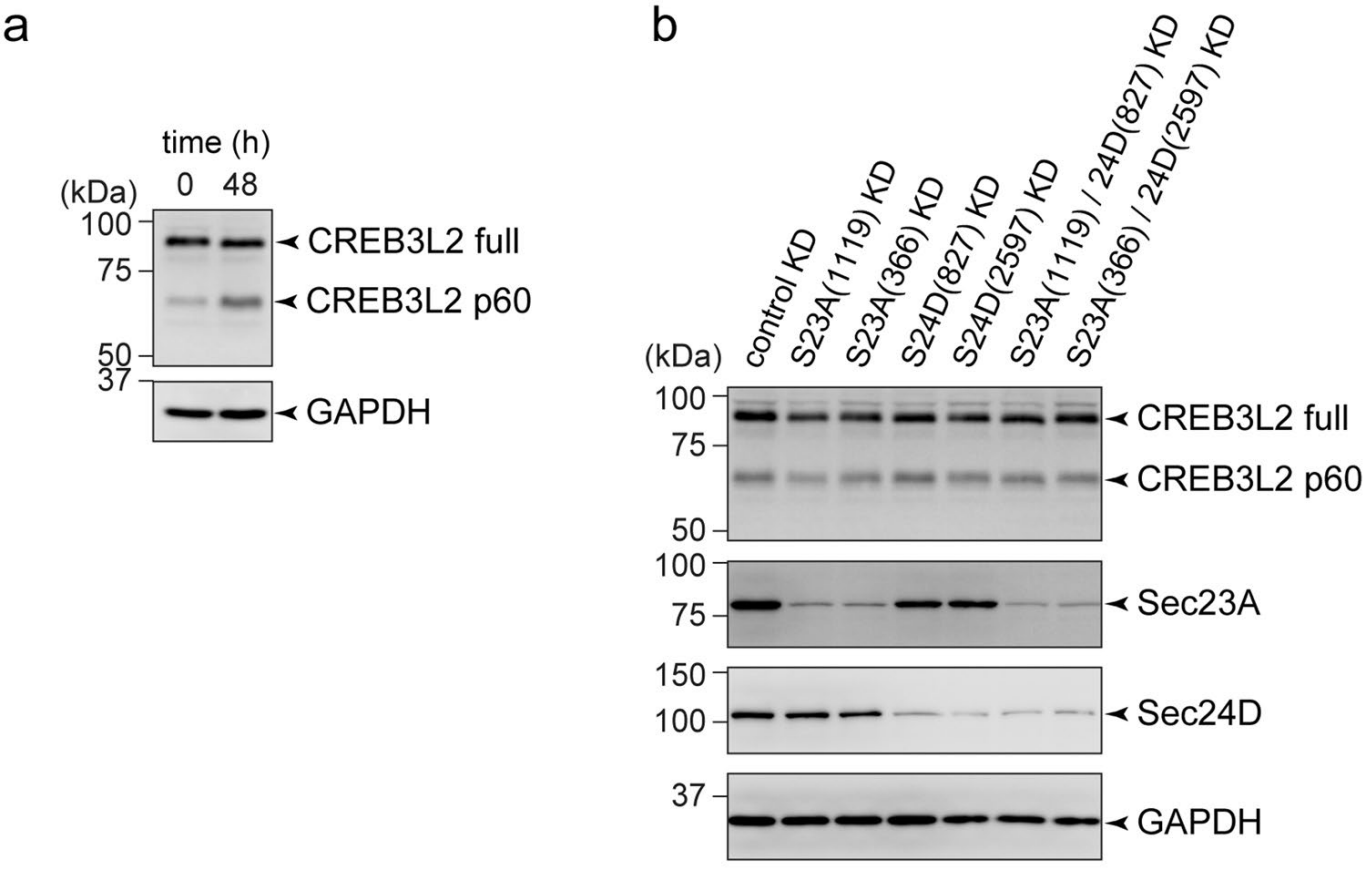
CREB3L2/BBF2H7 transport from the ER to the Golgi does not depend on Sec23A and Sec24D. (**a**) LX-2 cells starved for 24 h with DMEM supplemented with 0.5% FBS were treated with 1 ng/ml TGF-β1 for the indicated times. Proteins were extracted and subjected to SDS-PAGE, followed by western blotting with anti-CREB3L2/BBF2H7 and anti-GAPDH antibodies. (**b**) LX-2 cells transfected with the indicated siRNA(s) were cultured for 24 h in DMEM supplemented with 10% FBS. After starvation for 24 h with DMEM supplemented with 0.5% FBS, the cells were treated with 1 ng/ml TGF-β1 and cultured for 48 h. Proteins were extracted and subjected to SDS-PAGE, followed by western blotting with anti-CREB3L2/BBF2H7, anti-Sec23A, anti-Sec24D and anti-GAPDH antibodies. Shown are representative immunoblots (*n* = 3). Uncropped original blots were shown in Fig. S3.

The above results indicated that impaired activation of HSCs upon Sec23A/Sec24D depletion depends on a factor(s) that is not secreted from the plasma membrane, but transported by a COPII-dependent process from the ER to the Golgi. One promising candidate that fulfils this condition is CREB3L2/BBF2H7. CREB3L2/BBF2H7 is an ER-synthesized transmembrane transcription factor, which is presumably transported from the ER to the Golgi upon activation, and is cleaved by S1P protease at the Golgi before translocating to the nucleus, where it functions as a transcription factor^7^. If so, this means that there is a positive feedback loop in which CREB3L2/BBF2H7 activation requires Sec23A/Sec24D-dependent ER-Golgi transport and in turn activated CREB3L2/BBF2H7 induces the expression of both Sec23A and Sec24D. Thus, we next investigated whether activated CREB3L2/BBF2H7 is transported to the Golgi via a Sec23A/Sec24D-mediated COPII-dependent pathway. To this end, we checked the amount of cleaved CREB3L2/BBF2H7 upon depletion of Sec23A and Sec24D. As shown in Fig. 5a, 48 h after TGF-β stimulation, the p60 form of the cleaved CREB3L2/BBF2H7 was significantly increased. Next, we investigated whether depletion of Sec23A and/or Sec24D affects the cleavage of CREB3L2/BBF2H7. Depletion of Sec23A, Sec24D or both had no significant effect on the cleavage of CREB3L2/BBF2H7, implying that CREB3L2/BBF2H7 relocation from the ER to the Golgi does not depend on Sec23A/Sec24D (Fig. 5b).

## Discussion

**Figure 6:**
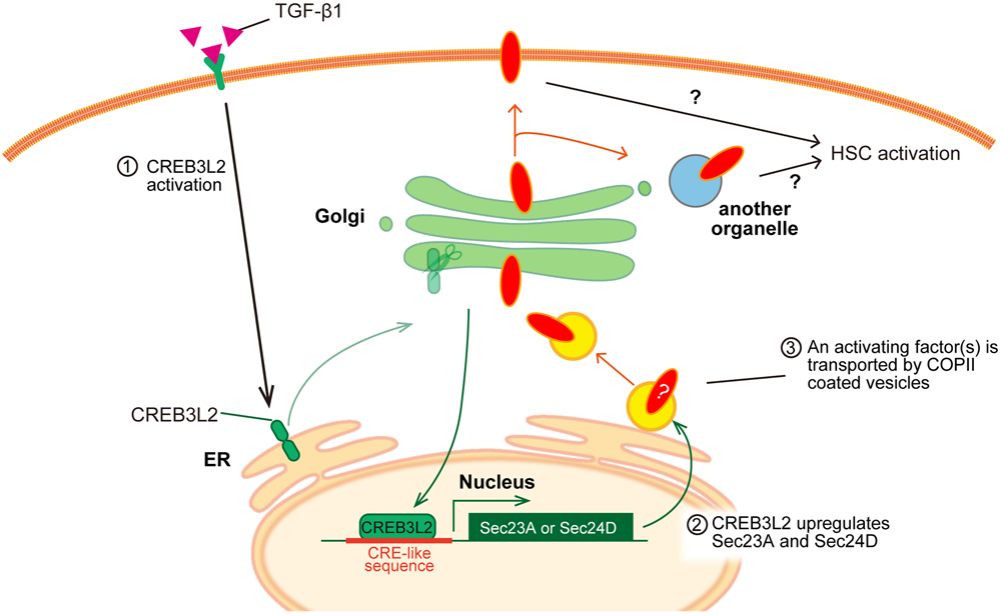
Model of the Sec23A/Sec24D-mediated HSC activation. (1) Cytokines such as TGF-β activate CREB3L2/BBF2H7. (2) Activated p60 CREB3L2/BBF2H7 upregulates the transcription of Sec23A and Sec24D. (3) An activated factor(s) is transported by a COPII-dependent process for HSC activation.

In this study, we showed that Sec23A and Sec24D are upregulated in an isoform-specific manner by the transmembrane transcription factor, CREB3L2/BBF2H7, during HSC activation. Moreover, we revealed that Sec23A/Sec24D-meditaed ER to Golgi trafficking is required for this activation. The identity of the factor(s) that needs to be transported for the activation remains to be revealed, but our analysis suggests that the transport of neither collagen I nor CREB3L2/BBF2H7 is regulated in a Sec23A/Sec24D-dependent manner. In addition, our data imply that the factor(s) is not secreted from the plasma membrane, but is transported from the ER to the Golgi for HSC activation. A summary of our findings is presented schematically in Fig. 6.

Although Sec23A depletion in primary rat HSCs inhibited collagen I secretion to the medium, knockdown of both Sec23A and Sec24D in LX-2 cells showed no effect on collagen I secretion. Primary rat HSCs are fairly undifferentiated when isolated and alter their morphology drastically during incubation. Conversely, LX-2 cells are potentially activated, but still possess the characteristics of undifferentiated HSCs. Moreover, the fact that most of the evidence for Sec23A/Sec24D involvement in collagen I trafficking comes from experiments on fibroblasts or *in vivo* data raises the possibility that their effects on collagen secretion is cell-type-specific^28–35^. Further experiments are required to resolve these issues, however, it is safe to conclude that collagen I secretion was not necessarily important for HSC activation when α-SMA expression was used as an index of activation in our system. α-SMA transcription has been reported to be regulated by several transcription factors including c-Myb and NF-κB^36^. Thus, it would be interesting to speculate that α-SMA expression is regulated by a transcription factor, whose activation requires Sec23A/Sec24D-dependent ER-to-Golgi transport. Otherwise, it may also be possible that a certain transcription factor(s) responsible for α-SMA induction is activated by a factor transported in a Sec23A/Sec24D-dependent fashion either directly or indirectly.

Recently, TANGO1 has been shown to be involved in HSC activation^37^. Maiers *et al*. have shown that TANGO1 expression was induced by TGF-β treatment in LX-2 cells via the unfolded protein response (UPR) pathway^37^. These findings seem contradictory at first to our data, demonstrating that TANGO1 expression was unchanged when Sec23A and Sec24D were significantly induced by TGF-β treatment in LX-2 cells. The major difference between these two experiments lies in the concentration of TGF-β. We used 1 ng/ml, while they treated the cells with 10 ng/ml. These findings prompted us to speculate that Sec23A and Sec24D are more sensitive than TANGO1 to cytokine stimulation, and their expression may be induced at first by activated CREB3L2/BBF2H7, which also requires either the UPR pathway or IGF signalling^25^. Thus, we propose that HSC activation originates from Sec23A/Sec24D-mediated transport, and then progresses with TANGO1-mediated collagen transport. We believe future studies will uncover the pathway and reveal the target(s) for preventing fibrosis.

## Methods

### Antibodies

Anti-Sec23A antibody was kindly provided by Dr. Bin Zhang (Cleveland clinic). Anti-cTAGE5, TANGO1 and Sec12 antibodies were raised and purified as described previously^16, 18, 19^. Anti-collagen I antibody (SP1.D8) was obtained from Developmental Studies Hybridoma Bank. Other antibodies were purchased from following companies: α-SMA (Abcam), CREB3L2/BBF2H7 (Atlas Antibodies), Sar1A (Novus Biological), β-actin (Chemicon), GAPDH (Chemicon), α-SMA-FITC antibody (SIGMA), Sar1B (Abnova), Smad2 (Cell signaling), pSmad2 (Cell signaling).

### Isolation and culture of primary rat hepatic stellate cells

Isolation of primary rat hepatic stellate cells was performed essentially described previously^11^. All experiments were performed with the approval of the animal experiment ethics committee at the University of Tokyo and according to the University of Tokyo guidelines for the care and use of laboratory animals. Properly anesthetized male Wister rats were *in situ* perfused with SC1 buffer (0.5 mM EGTA, 5.0 mM Glucose, 10 mM HEPES, 5.4 mM KCl, 0.85 mM Na_2_HPO_4_, 0.14 mM NaCl, 0.57 mM NaH_2_PO_4_, 4.2 mM NaHCO_3_, 0.02 mM Phenol Red) followed by SC2 buffer (3.8 mM CaCl_2_, 10 mM HEPES, 5.4 mM KCl, 0.85 mM Na_2_HPO_4_, 0.14 mM NaCl, 0.57 mM NaH_2_PO_4_, 4.2 mM NaHCO_3_, 0.02 mM Phenol Red) supplemented with actinase E, and then SC2 buffer with collagenase P. The perfused liver was excised and further digested in SC2 buffer supplemented actinase E, collagenase P and DNase I *in vitro*. The cell suspension was filtered to remove undigested materials followed by centrifugation at 600 × g for 10 min at 4 °C. The cell pellet was washed with GBSS buffer (1.5 mM CaCl_2_, 5.0 mM KCl, 5.5 mM Glucose, 0.22 mM KH_2_PO_4_, 1.0 mM MgCl_2_, 0.58 mM MgSO_4_, 0.42 mM Na_2_HPO_4_, 0.14 mM NaCl, 2.7 mM NaHCO_3_, 0.02 mM Phenol Red) and then suspended with GBSS buffer supplemented with DNase I. 0.72 volume of cell suspension was then mixed with 0.28 volume of Histodenz solution (8 g Histodenz in 28 ml of GBSS buffer without 0.14 mM NaCl). GBSS-B was then layered on the suspension and centrifuged at 1,500 × g for 15 min at 4 °C. Layer containing hepatic stellate cells was transferred into GBSS-B buffer and then centrifuged at 600 × g for 10 min at 4 °C. Isolated cells were cultured in DMEM supplemented with 10% fetal bovine serum.

### qRT-PCR

Total RNA was extracted from cells by RNAiso plus (Takara Bio Inc.). cDNA was prepared by ReveTra Ace qPCR RT Master Mix with genomic DNA Remover (Toyobo, Ltd.). qRT-PCR was performed with THUNDERBIRD SYBER qPCR Mix (Toyobo, Ltd.) using StepOnePlus RT-PCR System (Applied Biosystems) according to the manufacturer’s protocol. Primer sequences are listed in Table S1. Data were normalized with β-actin.

### Cell culture and transfection

LX-2 cells were cultured in DMEM supplemented with 10% fetal bovine serum. Lipofectamine RNAi max (Thermo Fisher Scientific) was used for transfecting siRNA.

Stealth select siRNAs were purchased from Thermo Fisher Scientific. Mission siRNA was purchased from SIGMA. siRNA sequences are listed in Table S2. For control siRNA, stealth RNAi siRNA negative control med GC duplex #1 (Thermo Fisher Scientific) or mission siRNA (SIGMA) control: AAUUCUCCGAACGUGUCACGUTT was used. The number in the parentheses represents the starting base pair of the target sequence.

### Sample preparation from cells and culture media

Primary rat HSCs were extracted with 1 × Laemmli sample buffer. Culture media of rat HSCs were precipitated with final 20% of ammonium sulfate for overnight at 4 °C. After centrifugation at 20,000 × g for 15 min at 4 °C, pellets were solubilized in 1 × Laemmli sample buffer. LX-2 cells extracted with 20 mM Tris-HCl pH 7.4, 100 mM NaCl, 1 mM EDTA, 1% TritonX-100 and protease inhibitors were centrifuged at 20,000 × g for 15 min at 4 °C. The supernatants were analyzed by Pierce 660 nm Protein Assay Reagent (Thermo Fisher Scientific) to normalize protein concentration for electrophoresis. Culture media of LX-2 cells were normalized by the protein concentration of cell lysate for electrophoresis. After western blotting, band intensities were quantified by Image Quant TL (GE healthcare).

### Luciferase assay

Effector protein expression plasmids were generated by subcloning wild type and N304K mutant CREB3L2/BBF2H7 into FLAG-tagged pCMV5 vectors (a gift from David Russell, UT Southwestern Medical Center, TX, USA). The firefly luciferase reporter plasmids were generated by replacing the original CRE sequence of the pGL4.29 vector with either 525 bp (wild type, mut1, mut2 and mut 1 + 2 indicated in Fig. 3c) or 205 bp of the promoter sequence upstream of human *Sec24D* (Promega). HEK293T cells were transfected with a reporter plasmid (0.8 μg), a reference plasmid pRL-TK (0.08 μg) carrying the *Renilla* luciferase gene under the control of HSV-thymidine kinase promoter (Promega) and an effector protein expression plasmid (0.8 μg), using Lipofectamine 2000 (Thermo Fisher Scientific). After 24 h, luciferase activities were measured using Dual-Luciferase Reporter Assay System (Promega) and a luminometer (BMG Labtech), according to the manufacturer’s protocol. Relative activity was defined as the ratio of firefly luciferase activity to that of *Renilla* luciferase.

### Data Availability

The data that support the findings of this study are available from the corresponding author upon request.

## Electronic supplementary material

Supplementary Information
