# Supplementary material for: CREB3L2-mediated expression of Sec23A/Sec24D is involved in hepatic stellate cell activation through ER-Golgi transport
Source: Sci Rep. 2017 Aug 11;7:7992. doi: 10.1038/s41598-017-08703-6 (PMC5554210; doi:10.1038/s41598-017-08703-6)
Supplement: Supplementary file 1 — Supplementary Information [file 41598_2017_8703_MOESM1_ESM.pdf]

Supplementary Information

CREB3L2-mediated expression of Sec23A/Sec24D is involved in hepatic stellate cell activation through ER-Golgi transport

Shotaro Tomoishi[1], Shinichi Fukushima[1], Kentaro Shinohara, Toshiaki Katada and Kota Saito*

Department of Physiological Chemistry, Graduate School of Pharmaceutical Sciences, University of Tokyo, 7-3-1 Hongo, Bunkyo-ku, Tokyo 113-0033, Japan

* Correspondence and requests for materials should be address to:
K. S. (ksaito@mol.f.u-tokyo.ac.jp)

[1]These authors contributed equally to this work.

Toshiaki Katada' s present address: Faculty of Pharmacy, Musashino University, Tokyo 202-8585 Japan

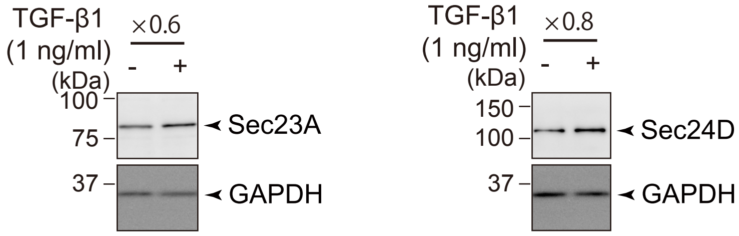

Figure S1. Sec23A and Sec24D are upregulated in LX-2 cells upon TGF-β1 stimulation.
The lysates used in Figure 2A were diluted as indicated and subjected to SDS-PAGE followed by western blotting with anti-Sec23A, anti-Sec24D and anti-GAPDH antibodies.

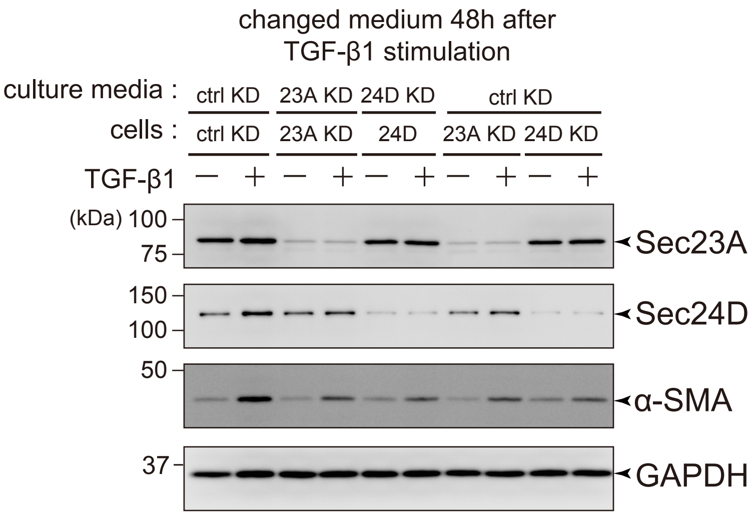

Figure S2. Sec23A/Sec24D-mediated HSC activation does not depend on secreted factors.
LX-2 cells transfected with the indicated siRNA(s) were cultured for 24 h in DMEM supplemented with 10% FBS. After starvation for 24 h with DMEM supplemented with 0.5% FBS, the cells were untreated or treated with 1 ng/ml TGF-β1 for 48 h. The culture medium was replaced with pre-cultured medium of cells treated with the indicted siRNA(s) and further cultured for 24 h. Proteins were extracted and subjected to SDS-PAGE, followed by western blotting with anti-Sec23A, anti-Sec24D, anti-α-SMA and anti-GAPDH antibodies. Shown are representative immunoblots (n = 3).

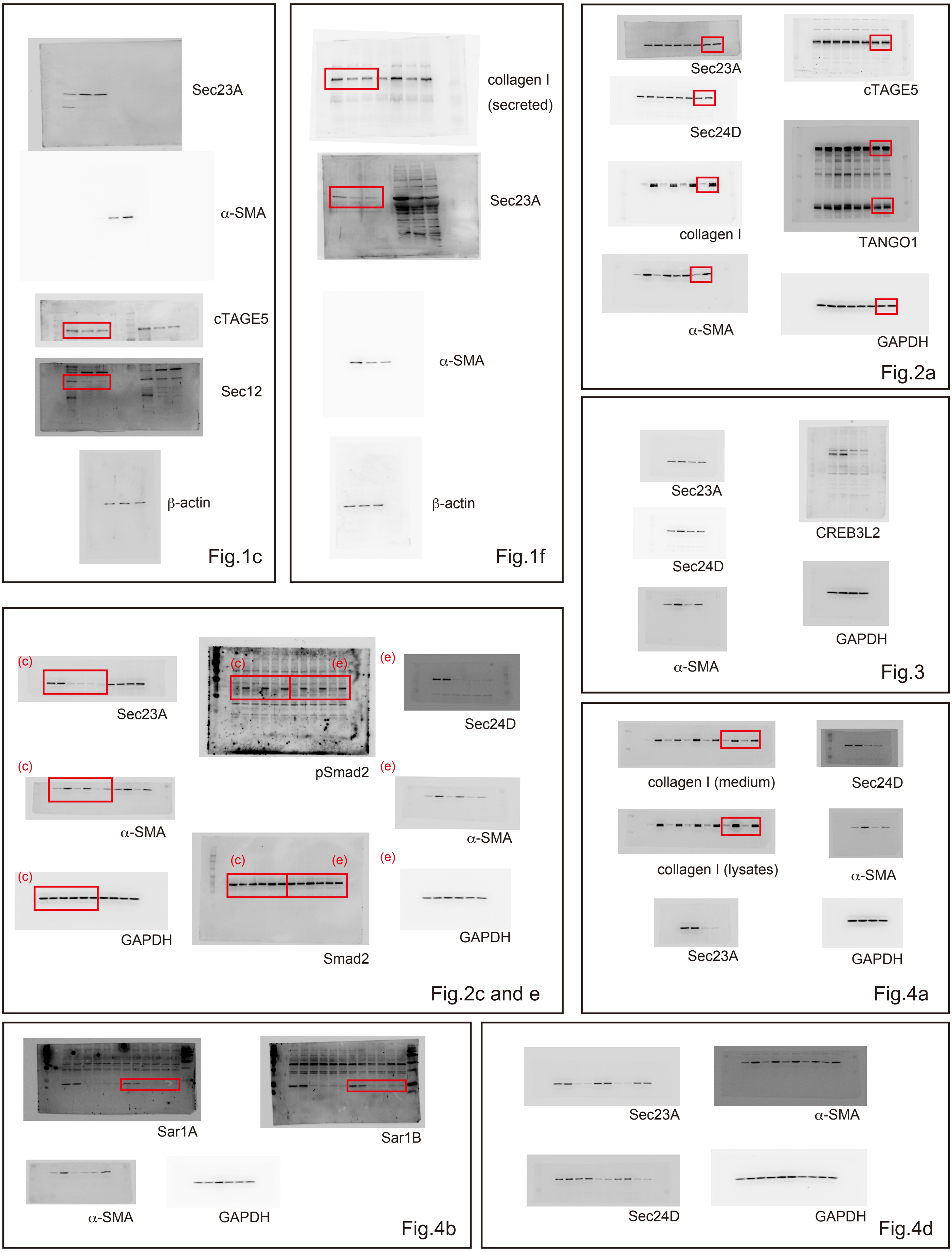

Figure S3. uncropped western blots.

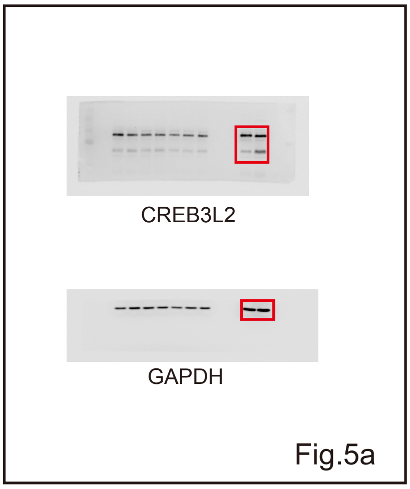

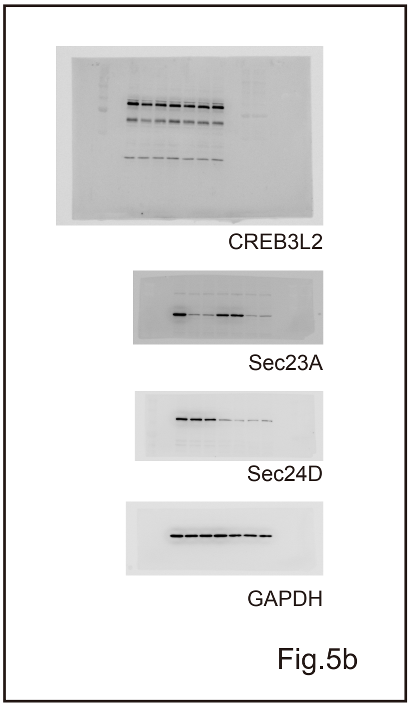

Figure S3. uncropped western blots.

Rat

Gene	RefSeq acession number	Primers	
		Sense	Antisense
β-actin	NM_031144	CCCGCGAGTACAACCTTCT	CGTCATCCATGGCGAACT
collagen I (α2)	NM_053356	CCTGGAGAACCTGGTCTCAT	GGCCAACATTTCCAGGAG
α-SMA	NM_031004	TGCCATGTATGTGGCTATTCA	ACCAGTTGTACGTCCAGAAGC
TANGO1	XM_001064186	AGCCGATGAAAAGGTGGTT	TCAATTCGCCGCTTGTAAGT
ctage5	XM_006225775	GGGACTTCCCTGGACCAC	CCCTTGGTGCGTACACAGT
Sar1A	NM_001007739	GGGCAAACCACAGGAAAG	CACTGCACATGAACACTTCCA
Sar1B	NM_001009622	CAGCACGTCCCAACACTACA	AAACGTCATGCCAGCAATAGT
Sec12	NM_001170708	TCGGATAGACCCCAAGACTG	CACCCGCTGATCTGCTCTA
Sec23A	NM_001105732	GGAGATGAAGTGCTGCCCTA	TTGAAAGAGTCACCCATTACCAT
Sec23B	NM_001108593	CTGGTCAACCTCAGGAGCA	TCTTGTGAACGGGCTGTAGA
Sec24A	NM_001105780	GACAGTATTCCGATTTGGCTTC	TGTGCTGATGGTGGTACGAG
Sec24B	NM_001106474	TCAGCTTCCGATGATGAGG	GCGCTGCTTGTGGTAGAAG
Sec24C	NM_001109456	GCCTTGACGTTAGGAACGAA	CATCCCCGATTTGATCTCAC
Sec24D	XM_003749389	GACCGTGCTGAAGAGGCTAC	GAAACCAACTCGAATTGCAGA
Sec13	NM_001006978	GTGACTGGGTTCGAGACGTT	GGCGTCATCACAAGTCCAA
Sec31	NM_033021	AGCCTGTCCCTCTGACACAC	AACGTTGCCATGCATTATGA
Bet3	NM_001008376	TTTCGGGAAACTGCTGATG	CCAGTTGGTGATGCTTGGA
CK1δ	NM_139060	GGCTCCTTCGGAGACATCTA	TGAGGATGTTTGGTTTTGACA
PP6	NM_133589	CGCAGGTGTACGGATTTTATG	TGAGCATGTCAAAAACTTTGGT
p125	NM_001134859	TGCTTGACTCTCTGAATCTTGAA	CCGTGCCTAGAACCACATTC
PCTAIRE	NM_031077	AGGGTCGCAATCGGATCT	CCTTTAGTGCAAATATGGAAGTAGTG
USP10	NM_001034146	GGTGGCAAGAGAGTCTGTCC	GTCACTCTACGGCTGACTTCAA
Dynactin1	NM_024130	GCCAATCCCAGATCCAAG	TTGAAGCAGAAGAATCAGGAG
KLHL12	NM_153730	TATGGCCACCAAGCGTTC	CCCACCACATAAATATGGTCATT
Sedlin	NM_001024965	GTCTGGAAGCTTCTACTTTGTAATTG	ACTGGTTCAGATGACGGTGA

Table S1. Sequences of primers used in this study.

Rat

siRNA	sequence
Sec23A (2138)	CCCGCGAGTACAACCTTCT
Sec23A (1358)	CCTGGAGAACCTGGTCTCAT

Human

siRNA	sequence
Sec23A (1119)	GGGUGAUUCUUUCAAUACUUCCUUA
Sec23A (366)	GCGUGGUCCUCAGAUGCCUUUGAUA
Sec24D (827)	GAGCCAGCAGAGGAGGACAAGUUUA
Sec24D (2597)	GAGCAUACCAGAGACAGCUGGUCAU
CREB3L2	GAGUCUUGUUCAACUGAGATT
Sar1A (150)	GGGCCAACAUGUUCCAACACUACAU
Sar1A (269)	UCCCAGCAAUUAAUGGGAUUGUCUU
Sar1B (162)	CCCAACAUUACAUCCCACUUCCGAA
Sar1B (495)	GAAAGAACUGAAUGCCCGACCCUUA

Table S2. Sequences of siRNAs used in this study.
